# Pulsed interleaved excitation-based line-scanning spatial correlation spectroscopy (PIE-lsSCS)

**DOI:** 10.1038/s41598-018-35146-4

**Published:** 2018-11-13

**Authors:** Xiang Gao, Peng Gao, Benedikt Prunsche, Karin Nienhaus, Gerd Ulrich Nienhaus

**Affiliations:** 1 Institute of Applied Physics, Karlsruhe Institute of Technology, 76128 Karlsruhe, Germany; 2 Institute of Nanotechnology, Karlsruhe Institute of Technology, 76344 Eggenstein-Leopoldshafen, Germany; 3 Institute of Toxicology and Genetics, Karlsruhe Institute of Technology, 76344 Eggenstein-Leopoldshafen, Germany; 4 Department of Physics, University of Illinois at Urbana-Champaign, Urbana, Illinois 61801 USA

## Abstract

We report pulsed interleaved excitation (PIE) based line-scanning spatial correlation spectroscopy (PIE-lsSCS), a quantitative fluorescence microscopy method for the study of dynamics in free-standing lipid bilayer membranes. Using a confocal microscope, we scan multiple lines perpendicularly through the membrane, each one laterally displaced from the previous one by several ten nanometers. Scanning through the membrane enables us to eliminate intensity fluctuations due to membrane displacements with respect to the observation volume. The diffusion of fluorescent molecules within the membrane is quantified by spatial correlation analysis, based on the fixed lag times between successive line scans. PIE affords dual-color excitation within a single line scan and avoids channel crosstalk. PIE-lsSCS data are acquired from a larger membrane region so that sampling is more efficient. Moreover, the local photon flux is reduced compared with single-point experiments, resulting in a smaller fraction of photobleached molecules for identical exposure times. This is helpful for precise measurements on live cells and tissues. We have evaluated the method with experiments on fluorescently labeled giant unilamellar vesicles (GUVs) and membrane-stained live cells.

## Introduction

Fluorescence fluctuation spectroscopy (FFS) comprises a wide and still expanding array of techniques that probe the dynamics of fluorescent molecules by analyzing temporal and spatial correlations of their fluorescence intensity^1^. These approaches started in the 1970s with the introduction of fluorescence correlation spectroscopy (FCS) ^2–4^, a powerful tool for measuring biomolecular dynamics in 2D or 3D fluidic systems (*e.g*., membranes, solutions, cells, organisms) over wide ranges of time. Various parameters can be quantified, including local fluorophore concentrations, translational and rotational diffusion coefficients, chemical rate and equilibrium coefficients, and the internal photodynamics of the fluorophores can also be studied^1^. FCS is based on the analysis of time correlations in intensity fluctuations of fluorescence light emanating from a tiny volume of typically 1 fl by using a conventional confocal microscope with diffraction-limited optics^5^. The observation volume can be further decreased by combining FCS with stimulated emission depletion (STED)^6^. Conventional, or static FCS, for which the observation volume is kept fixed at a particular location in the sample, has serious drawbacks for studies of the rather slow diffusional dynamics in biomembranes: The infrequent appearance of fluorophores in the focal spot poses sampling problems and their long retention in the focus enhances the likelihood of photodestruction. Scanning the focus in the plane of the membrane during FCS data acquisition alleviates such problems since the observation volume is distributed over a wider region^7,8^. Scanning FCS is excellent for studying planar membranes on solid supports but less well suited for free-standing membranes, *e.g*., plasma membranes of living cells. This is because biomembranes are extremely thin (~5 nm) and soft, and perform significant spatial fluctuations in the absence of a solid support. The resulting displacements relative to the observation volume give rise to considerable intensity fluctuations superseding those from motions within the membrane and so spoil the FCS experiment. Line-scanning FCS (lsFCS), in which the observation volume is raster-scanned along a line perpendicularly through the membrane, offers a solution to this problem^9,10^. In this approach, the intensities of those pixels during which the focus intersects the membrane are integrated and aligned to a common time for correlation analysis. Typically, lines are scanned in the focal plane by using relatively slow galvanometer scanners. Accordingly, lsFCS has a lower time resolution (~1 ms) than static FCS and requires longer data acquisition times because the focus crosses the membrane only during a small fraction of the total duration of the measurement. Photobleaching can still pose problems because the same small region of the membrane is continuously exposed to the excitation light during line scanning^8,11^. In lsFCS, dual-color correlation analysis can be implemented simply by using continuous-wave lasers and line sweeps with alternating colors. Alternatively, quasi-simultaneous pulsed interleaved excitation (PIE) with picosecond-pulsed lasers permits dual-color excitation in a single sweep and, thus, offers two-fold higher time resolution.

Raster image correlation spectroscopy (RICS) is an alternative method to study diffusional dynamics, especially in live cells and tissues. It exploits spatio-temporal correlations of fluorescence intensity fluctuations from raster-scanned images^12–14^. A key advantage of RICS over FCS is that the region of interest (ROI) can be selected in the image after its acquisition. Because pairs of pixels are correlated over an entire ROI, the spatial resolution of RICS is worse than the one of static FCS but can be reduced to ~300 nm by combining RICS with STED nanoscopy^15^. RICS has been widely applied to investigations of molecular motions within cell membranes^15,16^. Fluorescence correlation methods have been further enhanced in a variety of ways, *e.g*., by using dual-color excitation with continuous-wave^17^ or pulsed lasers^18,19^.

Here, we introduce PIE-based dual-color line-scanning spatial correlation spectroscopy, abbreviated PIE-lsSCS, and show that this technique is excellent for studying molecular dynamics within free-standing membranes. PIE affords crosstalk-free dual-color cross-correlation analysis, and line scanning allows us to avoid problems due to membrane movements. The analysis of intensity pair correlations between many pixels improves the sampling of sparse and slowly moving molecules, and also lowers the local photon flux, resulting in a reduced fraction of photobleached molecules in comparison to lsFCS.

## Results

### PIE-based dual-color line-scanning SCS

**Figure 1:**
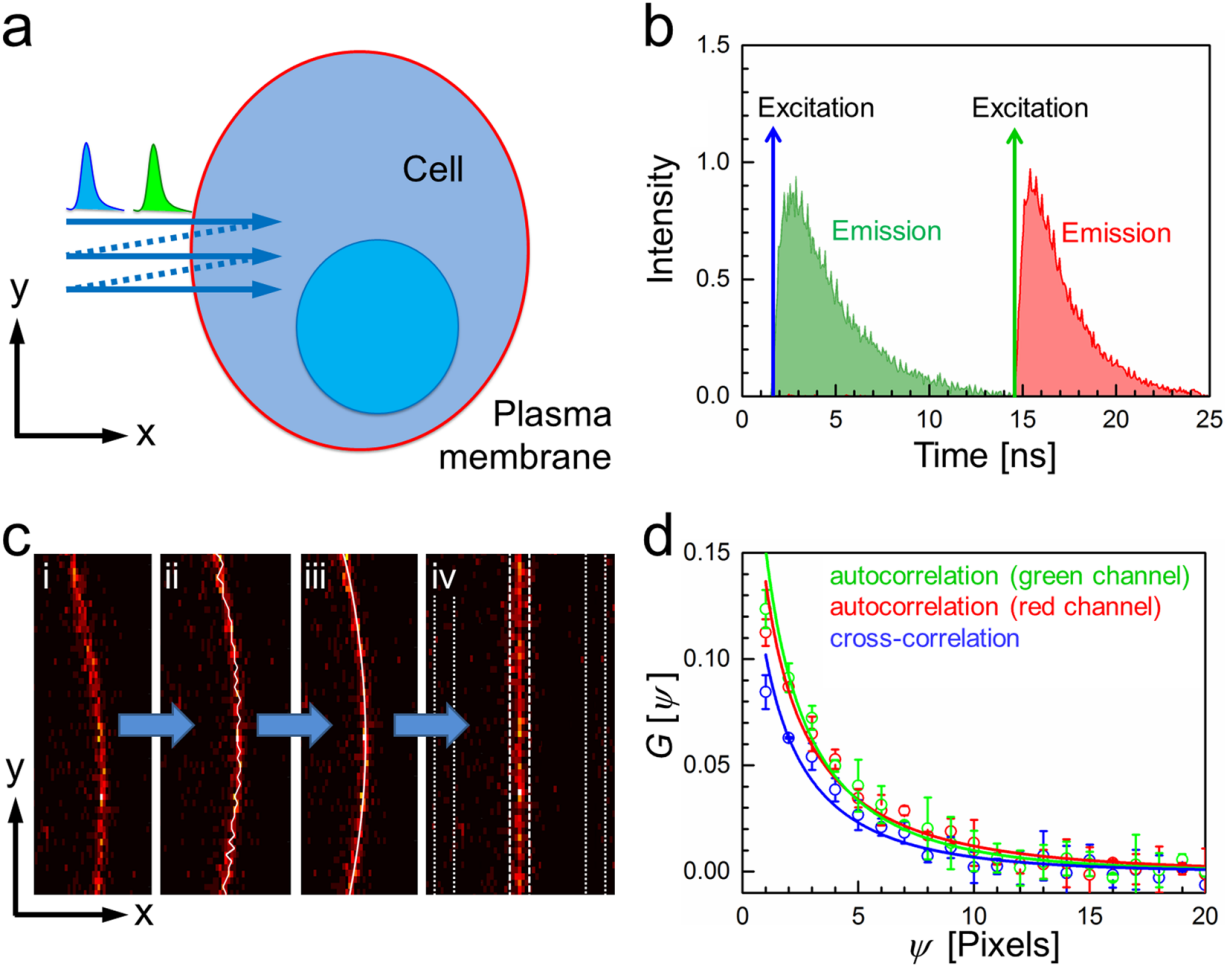
Principle of PIE-lsSCS. (**a**) A line is scanned multiple times along the *x*-axis perpendicular to the membrane (arrow), each time slightly displaced along the *y*-axis. (**b**) Two-color excitation and detection, with laser pulses and fluorescence emission interleaved in time. (**c**) In the recorded *xy*-images (i), the membrane location is identified for each line, *i*, in the following way: The membrane position *x*_i,raw_ is coarsely determined as the position of the pixel with the maximum intensity (ii). All *x*_i,raw_ values within one image are then fitted with a sixth-order polynomial, yielding refined membrane positions, *x*_i_ (iii). Each membrane position *x*_i_ is readjusted to a common origin, and a ROI with width 2*ω*_0_ in the *x*- direction around the common origin is selected for the following RICS analysis (iv). Here, *ω*_0_ is the lateral 1/e^2^ focus extension for 640-nm light. Dashed and dotted lines mark the membrane emission strip and the two strips used for background determination, each of them being five pixels wide. (**d**) Exemplary spatial autocorrelation (green and red) and cross-correlation (blue) curves measured on GUVs labeled with a double-stranded DNA carrying a cholesterol moiety for membrane attachment and two fluorophores (see Methods).

In this technique, fluorescence is excited by two spatially overlapped, focused laser beams performing repetitive, perpendicular line scans through a free-standing vertical membrane (along the *x*-axis in Fig. 1a). Typical parameters are as follows: Each scanned line consists of ~100 pixels separated by *δ*_x_ = 100 nm, with a pixel dwell time of 10–40 µs. Typically, 40 lines are scanned successively, with each new scan line being displaced from the previous one by several ten nanometers along the *y*-axis. Notably, scanning speed and pixel size along the *y*-axis, *δ*_y_, need to be carefully chosen to match the diffusional dynamics^12^. Thus, taking 128 × 40 pixels with *δ*_x_ = *δ*_y_ = 100 nm and pixel dwell time of 35 µs, a rectangular region of ~10 µm × 4 µm, intersected by the target membrane, is raster-scanned within 175 ms. This procedure is repeated up to ~1000 times to collect sufficient statistics. Each of the two laser sources generates a pulse train with one 100-ps pulse every 25 ns. Both pulse trains are mutually shifted in time by 12.5 ns, yielding two time windows during which the emission in the green and red channels decays (Fig. 1b). By using time-correlated single photon counting (TCSPC), the arrival time with respect to the excitation pulse is stored for each detected photon. Therefore, photons can be assigned unambiguously to the correct color channel and crosstalk is avoided. For each scanned line, an emission peak appears during an *x*-scan whenever the observation volume crosses the membrane. Its position can be identified and readjusted to a common origin to remove membrane curvature and dynamics (Fig. 1c). Notably, to determine the membrane location even more precisely, we could calculate running averages of 2*n* + 1 (*e.g*., *n* = 11) consecutive *xy*-images from a set of ~1000 to improve the statistics of image *n* + 1 because membrane displacements within such a small subset (but not in the entire set!) are negligible. Next, we subtract background, determined as an average of the intensities in strips of 5 pixels to the left and right of the 5 pixels wide strip containing the emission from the membrane (Fig. 1c). Before computing correlation functions, the background-corrected, 5 × 40 pixels membrane sub-images are further processed. Immobile structures are removed by subtracting, pixel by pixel, the average over the corresponding pixels in the entire set of *xy*-images from each pixel intensity and adding the average over all pixels from all images^20,21^. We normalize each *xy*-frame by dividing it by its average intensity, so that spatial correlation functions from many *xy*-frames can easily be averaged. Finally, we sum over the 5 pixels along the *x*-axis that contain membrane signals. In the end, these procedures reduce the 2D *xy*-image to a 1D vector containing 40 membrane intensity values along the *y*-dimension, corrected for background and the immobile fraction. Subsequently, these 40 intensity points or a subset thereof are spatially correlated (*vide infra*). In principle, other patterns of scanning trajectories could also be implemented depending on the problem at hand.

For spatial correlation analysis of a 2D image in two colors (*i, j*), spatial second-order autocorrelation (*i,i; j,j*) and cross-correlation (*i* ≠ *j*) functions are calculated from the intensity traces along the two scanning directions (*x* and *y*)^13^,

1 $${G}_{i,j}(\xi ,\psi )=\frac{\langle {I}_{i}(x,y){I}_{j}(x+\xi ,y+\psi )\rangle }{\langle {I}_{i}(x,y)\rangle \langle {I}_{j}(x+\xi ,y+\psi )\rangle }-{\rm{1}},$$

where *I*(*x*, *y*) and *I*(*x* + *ξ*, *y* + *ψ*) represent the pixel intensities at positions (*x*, *y*) and (*x* + *ξ*, *y* + *ψ*); the angular brackets indicate averages over all pairs of pixels in a selected ROI. Importantly, the spatial displacements *ξ* and *ψ* between pixels correspond to specific lag times resulting from raster scanning with fixed pixel and line dwell times. In this work, we use the *x*-axis only for compensation of membrane movements and spatially correlate the integrated membrane intensities along the *y*-axis (Fig. 1a). We note, however, that this 1D concept can be extended to 2D by collecting *z*-stacks of *xy*-images, yielding *yz*-images for 2D spatial correlation analysis (lsRICS). For a single *xy*-image, we use a 2D diffusion model with a single scanning dimension, so that

2 $${G}_{i,j}(\psi )={G}_{{diff}}(\psi )\cdot {G}_{{scan}}(\psi ),$$

with a diffusion term

3 $${G}_{{diff}}(\psi )=\frac{1}{\langle N\rangle }{(1+\frac{4D\psi {\tau }_{{\bf{l}}}}{{\omega }_{0}^{2}})}^{-1/2}{(1+\frac{4D\psi {\tau }_{{\bf{l}}}}{{z}_{0}^{2}})}^{-1/2},$$

and a scanning term

4 $${G}_{{scan}}(\psi )=exp(-\frac{{(2\psi {\delta }_{y})}^{2}}{2({\omega }_{0}^{2}+4D\psi {\tau }_{l})}).$$

The amplitudes of the autocorrelation functions are inversely proportional to the average number of fluorophores, 〈*N*〉, in the elliptically shaped observation area given by *πω*_0_*z*_0_. Here, the lateral (*ω*_0_) and axial (*z*_0_) extensions of the observation area are defined by the spatial separation of two points along the *x*- and *y*-axes with an intensity ratio of 1/*e*^2^; they need to be carefully determined by calibration experiments (see Methods) to extract precise area densities. Notably, for dual-color cross-correlation analysis, the effective values of *ω*_0_ and *z*_0_ are the root-mean-square averages of these parameters for the two colors. The line dwell time is given by *τ*_l_, and the spatial decay of the correlation yields the diffusion coefficient, *D*. Figure 1d shows examples of autocorrelation curves from the green and red color channels as well as their cross-correlation (blue).

### Measurements on GUVs labeled with two separate fluorophores

**Figure 2:**
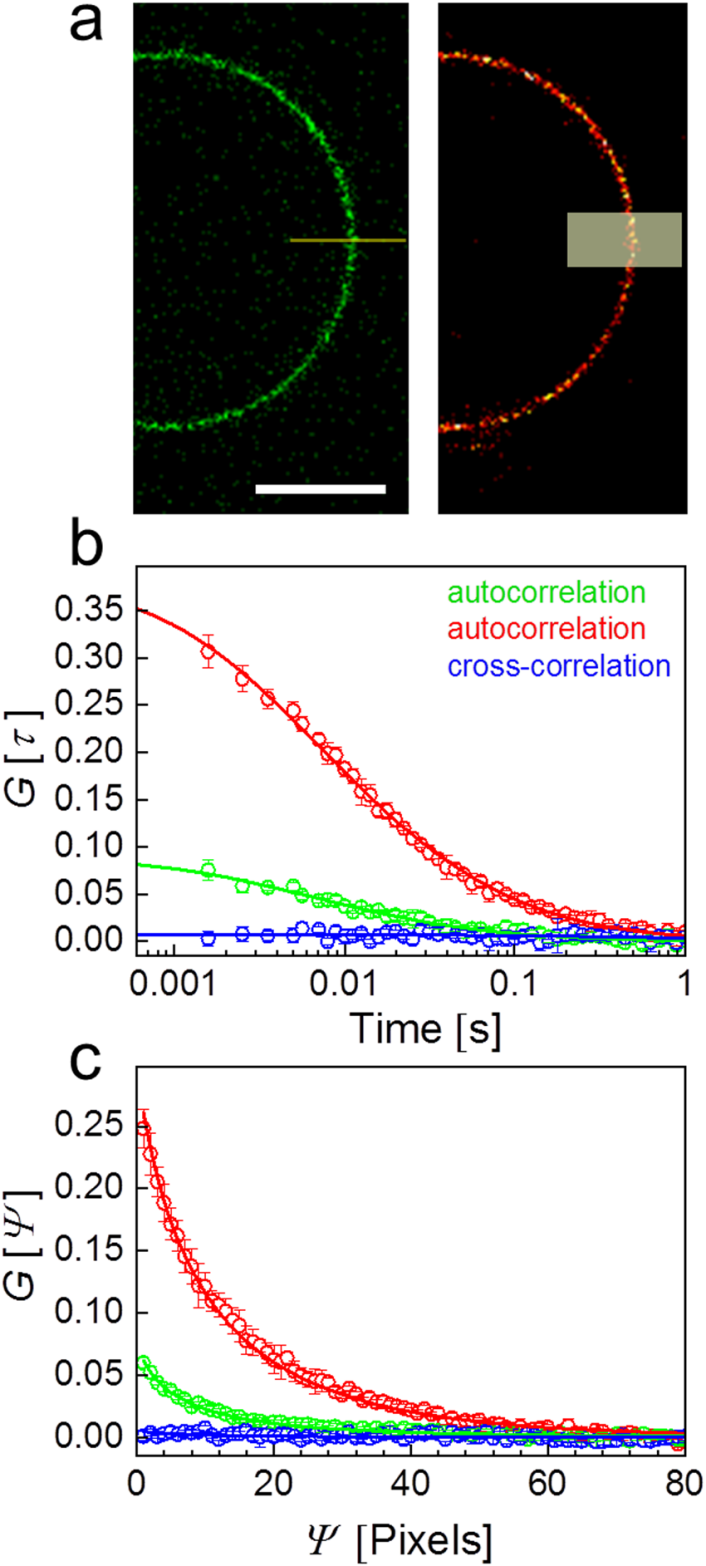
PIE-lsFCS and PIE-lsSCS experiments on DOPC/cholesterol GUVs labeled with DiI and Atto647N-DPPE. (**a**) Images of GUVs in the green and red channels, depicting the scanning schemes of lsFCS (left) and lsSCS (right); the horizontal line and the semi-transparent rectangle indicate the scanned regions, respectively. Scale bar, 15 µm. (**b**) lsFCS and (**c**) lsSCS correlation data (symbols) calculated from intensity time traces; error bars represent standard deviations from three independent measurements in the same region. Green and red colors: Autocorrelation functions in the green and red channels, respectively; blue: cross-correlation. Lines show fits with a 2D diffusion model.

We started our evaluation of lsSCS measurements on giant unilamellar vesicles (GUVs) made from 1,2-dioleoyl-sn-glycero-3-phosphocholine (DOPC) and cholesterol^22^, which were fluorescently labeled with DiI and Atto647N-DPPE. A rectangular area (10 µm × 4 µm, with its long axis crossing the membrane perpendicularly) was scanned with 100 × 200 pixels (pixel dwell time: 10 µs) 1000 times for a total measurement time of 200 s. For direct comparison, we also took lsFCS data for 300 s on the very same sample. The scanning schemes are depicted schematically in Fig. 2a. The DiI and Atto647N fluorophores were excited with 561 and 640 nm laser light at 2 µW in both experiments. The measured lsFCS time autocorrelations (Fig. 2b) and the lsSCS spatial autocorrelations (Fig. 2c) are in excellent agreement with fits with a model function based on 2D diffusion within the membrane (for lsSCS, see equations (2–4), for lsFCS, we refer to ref.^11^). Notably, the cross-correlation amplitude is zero, showing that DiI and Atto647N-DPPE diffuse independently in the membrane. Moreover, because channel crosstalk would produce an artificial cross-correlation (see Supplementary Information, Fig. S1), this result also attests to the perfect crosstalk suppression thanks to PIE. The area densities and diffusion coefficients obtained from fits to the lsFCS and lsSCS autocorrelations are compiled in Table 1. The good agreement between the results from both methods shows that lsSCS is a reliable technique for quantifying diffusion in free-standing 2D membranes. With the scan parameters used here, fluorophores are on average exposed to roughly 13-fold less photons during the lsSCS experiment with respect to lsFCS due to the larger effective observation region and the different overall measurement times. In lsSCS, 640-nm irradiation is spread over a roughly rectangular area of 100*δ*_y_ × 2*z*_0_ = 8.5 µm^2^, whereas lsFCS involves irradiation of an elliptical area, *πω*_0_*z*_0_ = 1.37 µm^2^. As a result, the fraction of photobleached molecules in the observation area is decreased. Here, we used fairly robust synthetic dyes with excellent photostability to label our GUVs. Biological samples such as live cells and tissues, however, are often labeled with genetically encoded fluorescent proteins of the GFP family^23^, which are photochemically less stable. Moreover, fluorophore densities are not well controllable in these experiments and often very low. Significant photobleaching causes an artificial decrease of the concentrations (area densities) and thus gives rise to systematic errors. Therefore, the reduced local photon flux offered by lsSCS is advantageous when using such samples (*vide infra*).

**Table 1:** Fit parameters of autocorrelation functions from lsFCS and lsSCS measurements on GUVs labeled with two independently diffusing fluorophores.

Method	Label	Area density/µm^−2^	D/µm^2^ s^−1^
lsFCS	DiI	11.2 ± 0.6	5.0 ± 1.0
	Atto647N-DPPE	2.6 ± 0.1	6.8 ± 0.9
lsSCS	DiI	13.5 ± 1.1	6.2 ± 1.5
	Atto647N-DPPE	3.4 ± 0.1	5.7 ± 0.3

Errors indicate uncertainties given by the non-linear least-squares fitting routine.

### PIE-lsSCS measurements on GUVs labeled with a dual-color DNA probe

**Figure 3:**
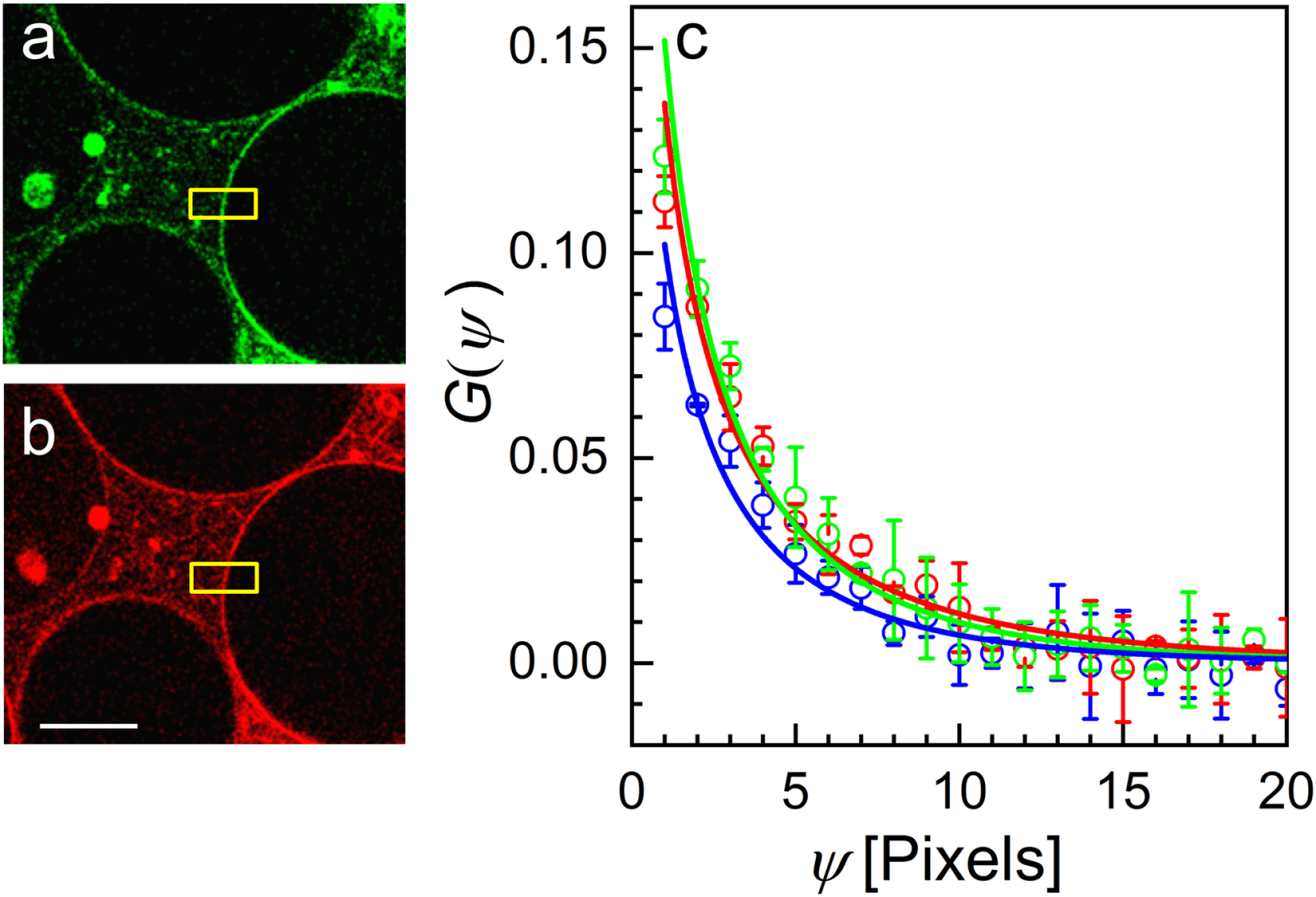
PIE-lsSCS on GUVs labeled with a dual-color probe. A double-stranded DNA carrying a bound cholesterol moiety for membrane insertion at the end of strand 1 and Atto488 and Atto565 fluorophores on strand 2 was used for dual-color labeling. Exemplary confocal images are depicted in the (**a**) green and (**b**) red channels; scale bar, 15 µm. (**c**) Experimental autocorrelation (green and red symbols) and cross-correlation (blue) data; error bars represent standard deviations from three independent measurements in the same region. Lines are fits with a 2D diffusion model.

Next, we investigated lateral diffusion of double-stranded DNA labeled with two fluorophores. For insertion into the GUV membrane, one strand carried a cholesterol moiety at the 3′ end; the complementary strand was labeled with Atto488 and Atto565 at the 5′ and 3′ ends, respectively, to ensure an optimal cross-correlation between the two color channels. For dual-color excitation, we used 470-nm (14 µW) and 561-nm lasers (6 µW). Figure 3a,b shows GUV images in the two color channels with the regions chosen for lsSCS analysis marked in yellow. The two autocorrelations and the cross-correlation are plotted in Fig. 3c together with the fitted model functions. From the fits, we obtained area densities from the autocorrelation functions in the green and the red channels and the cross-correlation function of 5 ± 1 µm^−2^, 4 ± 1 µm^−2^, and 3 ± 1 µm^−2^; the corresponding diffusion coefficients were 8.4 ± 1.8 µm^2^ s^−1^, 11.7 ± 2.4 µm^2^ s^−1^, and 8.1 ± 2.0 µm^2^ s^−1^, respectively. The parameters obtained from the autocorrelation functions of the two channels are identical within the error, and the diffusion coefficient from the cross-correlation also agrees with the other two values. The amplitude of the cross-correlation is slightly less than the mean of the two autocorrelation amplitudes, which may result from a small displacement between the two observation volumes. As an alternative way of analysis, we also performed a global fit of all three correlation functions by taking *D* as a shared parameter, yielding *D* = 8.4 ± 1.8 µm^2^ s^−1^.

### PIE-lsSCS measurements on live NCI-H1703 cells

**Figure 4:**
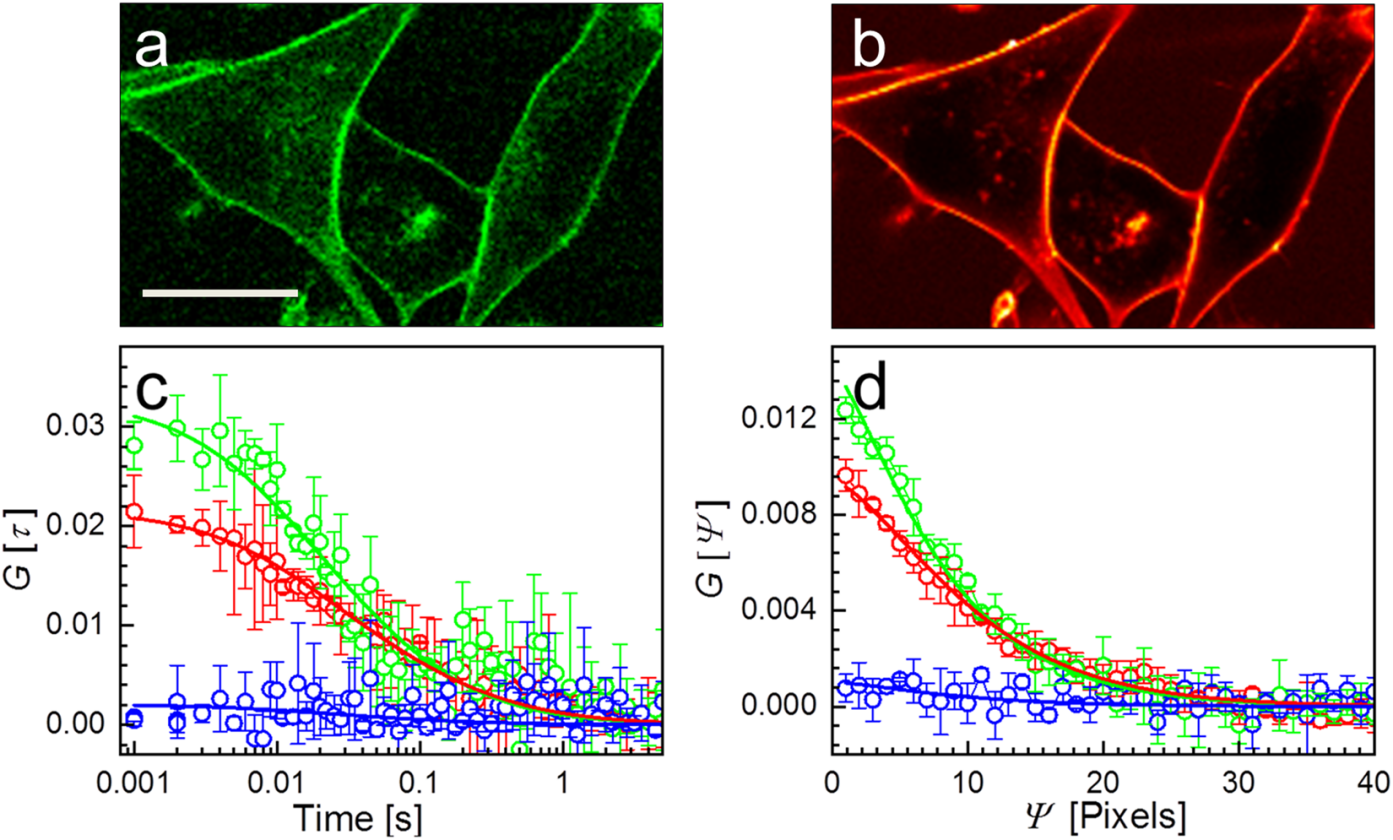
PIE-lsFCS and PIE-lsSCS on plasma membranes of NCI-H1703 cells with dual-color membrane staining. Confocal images of the H1703 cells in the (**a**) green and (**b**) red color channels. Cells were stained immediately before the experiment. Scale bar, 15 µm. (**c**) lsFCS and (**d**) lsSCS correlation data (symbols) calculated from intensity time traces; error bars represent standard deviations from three independent measurements in the same region. Lines are fits with a 2D diffusion model. Green and red colors: Autocorrelation functions in the green and red channels, respectively; blue: cross-correlations.

Figure 4a,b shows exemplary confocal images of NCI-H1703 cells in the green and red color channels. The cells were labeled with CellMask Green and CellMask Deep Red plasma membrane stains. For best comparison, we carried out dual-color lsFCS and lsSCS experiments with pulsed interleaved 640-nm (6 µW) and 470-nm (10 µW) excitation on the same cell. For lsFCS, we scanned a single line (10 µm) with 100 pixels perpendicularly crossing the membrane for a total duration of 300 s. For lsSCS, a rectangular area (10 µm × 2 µm, with its long axis crossing the membrane perpendicularly) was scanned with 100 × 100 pixels (pixel dwell time: 10 µs) 330 times, thus giving a total measurement time of 33 s. Compensation for immobile fractions was performed for subsets of 30 frames each to minimize problems due to possible cell movements^20^. The correlation curves obtained from the lsFCS and lsSCS experiments are shown in Fig. 4c,d, respectively. Both lsFCS and lsSCS data yielded cross-correlation amplitudes close to zero, indicating that the red and green fluorophores predominantly diffuse independently from each other within the membrane. Careful inspection shows very small cross-correlation amplitudes, however, which presumably arise from both dyes binding simultaneously to proteins or protein clusters in the membrane. The fit of the FCS data in Fig. 4c with a 2D diffusion model^11^ revealed diffusional correlation times, *τ*_D_, in the green and red channels of 10 ± 2 ms and 13 ± 3 ms, resulting in diffusion coefficients of the green and red species of 1.3 ± 0.5 µm^2^ s^−1^ and 1.7 ± 0.6 µm^2^ s^−1^, respectively. Fits of the lsSCS data in Fig. 4d with equations (2–4) gave diffusion coefficients of the green and red species of 1.81 ± 0.10 µm^2^ s^−1^ and 1.68 ± 0.13 µm^2^ s^−1^, respectively. Here, we report averages and standard deviations from three measurements on the same spot. For comparison, a subsequent experiment at three different locations yielded 1.34 ± 0.28 µm^2^ s^−1^ and 1.41 ± 0.15 µm^2^ s^−1^, respectively. The different averages and the greater standard deviations in the second experiment are likely caused by the heterogeneity of the plasma membrane giving rise to locally varying transport properties. The diffusion coefficients obtained by lsFCS and lsSCS agree with each other and are within the range of those reported for DiI-labeled cell membranes (0.8–3.2 µm^2^ s^−1^)^24^. The amplitudes of the lsFCS autocorrelation curves are larger than those of lsSCS. This discrepancy is predominantly due to the greater fraction of photobleached molecules during the lsFCS measurement. Thus, the fluorophore density is artificially decreased, which increases the autocorrelation amplitudes^8^. Here, the irradiation in lsSCS was spread over an extended, roughly rectangular area of 100*δ*_y_ × 2*z*_0_ = 5.8 µm^2^ (for red excitation). Furthermore, the measurement time of lsSCS was ninefold reduced, so that lsSCS allowed a reduction of the local light exposure by ~40-fold with respect to lsFCS.

## Discussion

In this work, we have introduced PIE-lsSCS as a new FFS variant and evaluated it against lsFCS. Both methods yielded, within the experimental error, identical area densities and translational diffusion coefficients of fluorescently labeled biomolecules undergoing diffusional motions within biological membranes. We emphasize that, in a SCS experiment, it is important to adjust the sampling parameters to the dynamics measured^12^. With line scanning of 100 pixels in 1 ms and the rather slow diffusion within membranes (~0.1–4 µm^2^ s^−1^)^24^, suitable step sizes are in the range of ~20–120 nm.

PIE-lsSCS offers shorter data acquisition times than lsFCS for comparable statistical uncertainty because sampling is more efficient in a larger region with multiple fluorophores than in a single spot^16^, so the probability of capturing slowly moving molecules is enhanced. These advantages obviously come at the expense of spatial resolution. Whereas this is not relevant for homogeneous model membranes such as the GUVs investigated here, cell membranes are heterogeneous and SCS analysis yields only average values over the observation region. The spatial resolution can, however, be significantly enhanced by combining lsSCS with STED microscopy^15^. If cytoskeletal structures locally confine movements or provide barriers that delay the dynamics within the cell membrane, there will be deviations of the data from the simple 2D diffusion model (equations 2–4)^25,26^. For such investigations, it can be advantageous to scan a larger *y*-region and to probe the dynamics in multiple ROIs within this image^16^.

2D diffusion in lipid bilayer membranes is comparatively slow, so photobleaching can locally deplete the pool of labeled molecules and lead to erroneous area densities. The enlarged irradiated area of PIE-lsSCS compared with lsFCS results in a smaller fraction of photobleached molecules at the effective observation site^3^. In an FFS experiment, significant photobleaching on timescales of the correlation decay will distort the correlation functions. Weaker photobleaching, on time scales greater than the correlation decay, however, does not change the shape of the autocorrelation functions but increases their amplitudes due to the slow disappearance of fluorescent molecules from the observation region during data collection. Therefore, the measured concentrations (area densities in membranes) are artificially lowered with respect to their true values. Thus, photobleaching can be a serious source of error in FFS experiments, and minimizing these effects is important, especially when working with less stable fluorophore labels such as GFP-like proteins.

We anticipate that PIE-lsSCS will be a versatile FFS tool for measuring dynamics in plasma membranes of live cells, tissues and perhaps even entire model organisms. Whereas the best spatial resolution can be achieved using single-spot lsFCS, averaging over larger regions inherent in the lsSCS method can be helpful for studies of heterogeneous biomembranes. In our own research, we will employ the technique for measuring the strengths of ligand-membrane receptor interactions in the Wnt signaling pathway^27,28^. In these experiments, receptors and ligands are labeled by using genetically engineered fusions with proteins of the GFP family. The reduced effects of photobleaching in the lsSCS experiment will be beneficial for determining precise number densities, from which we compute ligand-receptor equilibrium dissociation coefficients^11^.

In the future, we will further enhance the functionality of the PIE-lsSCS method. We have already mentioned the investigation of obstacles to diffusion within the membrane, but we can also explore the barrier function against transport across the plasma membrane or intracellular membranes^25,26^. This requires an extension of the spatial correlation analysis to the *x*-dimension, which we have so far only used to compensate membrane displacements. Another interesting extension will be to not only exploit TCSPC detection for better time resolution and crosstalk elimination within the PIE scheme, but to enhance the functionality of lsSCS by making use of the measured fluorescence decay information. For example, we can reduce (prompt) scattering noise by time gating^29^, *i.e*., by including only those photons in the analysis that are registered after the instrument response function has decayed. Furthermore, analysis of the entire fluorescence decay histograms will enable us to distinguish between species on the basis of significantly different fluorescence lifetimes. This approach was pioneered by Enderlein and coworkers for FCS and termed fluorescence lifetime correlation spectroscopy (FLCS^30,31^). More recently, Lamb and coworkers transferred this concept to raster images (raster lifetime image correlation spectroscopy, RLICS^19^). Lifetime analysis can be employed to increase the number of distinguishable fluorescent species, to quantify Förster resonance energy transfer (FRET) efficiencies, and it also permits efficient noise filtering^19^. This latter point is especially relevant for live-cell studies, *e.g*., of interactions between ligands and membrane receptors, in which the density of receptors fused with GFP-tags can be very low and cellular background is often quite high.

## Methods

### Confocal microscope for PIE-lsSCS and its alignment

**Figure 5:**
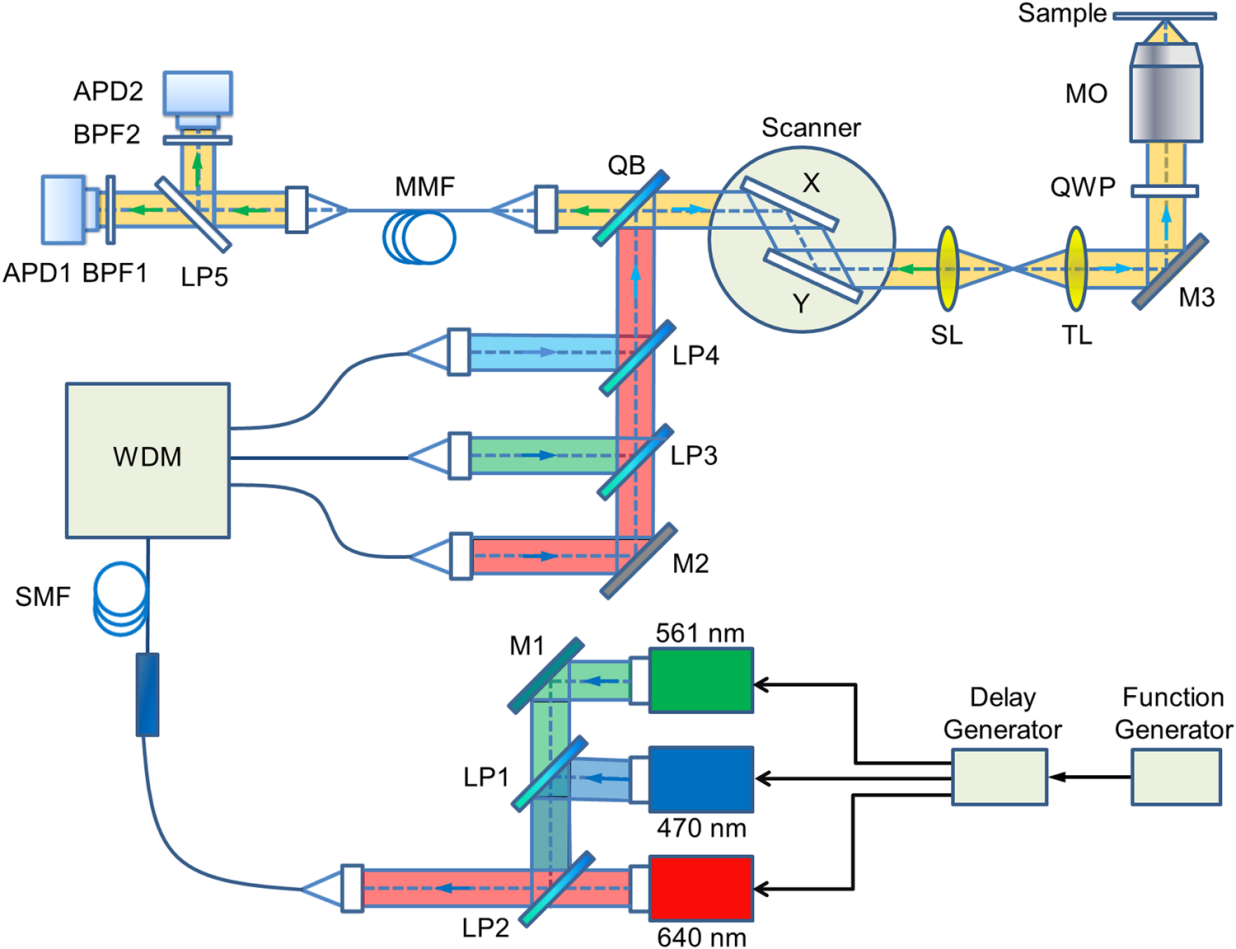
Schematic depiction of the confocal microscope used for PIE-lsSCS. Abbreviations: APD, avalanche photodiode; BPF: bandpass filter; LP, long-pass filters; M, mirrors; MMF, multi-mode fiber; MO, microscope objective; QB, quad-band mirror; QWP, quarter-wave plate; SMF, single-mode fiber; SL, scan lens; TL, tube lens; WDM: wavelength division multiplexer. Black arrows indicate laser timing controls.

Our home-built confocal microscope (Fig. 5) employs a commercial microscope frame (DMi8, Leica Microsystems, Mannheim, Germany). There are three picosecond pulsed lasers for excitation, emitting at 470 nm (LDH-P-C-470B, Picoquant, Berlin, Germany), 561 nm (PDL 561, Abberior, Göttingen, Germany) and 640 nm (LDH-P-C-640B; PicoQuant). The laser beams are coupled into a wideband fiber coupler (TW630R5A1, Thorlabs, Munich, Germany). To avoid axial misalignment of the foci, the combined beam is split into three paths via a wavelength dividing multiplexer (WDM, RGB26HA, Thorlabs), and further combined using longpass dichroic mirrors LP3 (575 nm longpass filter, Edmund Optics, Mainz, Germany) and LP4 (532 nm longpass filter, AHF, Tübingen, Germany). In each path, the position of the focal point is adjusted by changing the distance between the fiber end and the coupling lens. After reflection by quad-band mirror QB (zt 405/473/561/640 RPC, AHF), the excitation light passes through a laser scanner (Yanus V, Till Photonics, Gräfelfing, Germany), scan lens SL and tube lens TL, and is reflected by mirror M3 (BB1-E02, Thorlabs). A quarter-wave plate QWP (AQWP05M-600, Thorlabs) generates circular polarization so as to avoid polarization effects. The beam is finally focused into the sample by a water immersion objective MO (HCX PL APO W CORR CS 63×/1.2, Leica). For axial scanning, the objective can be moved with an accuracy of 0.5 nm by using a motorized linear actuator (M-122, Physik Instrumente, Karlsruhe, Germany) and a piezo actuator (P-720, Physik Instrumente).

The fluorescence emitted by the sample propagates back through QB and is coupled into a multi-mode fiber (MMF-IRVIS-62.5/125-0.245-L, OZ Optics, Ottawa, Canada) serving as a confocal pinhole of 1 Airy unit (for 640-nm light). Subsequently, the fluorescence is separated by the 555 nm longpass dichroic mirror LP5 (FF555-Di02, Semrock, Rochester, NY) into two color channels. Photons are passed through bandpass filters BPF1 and BPF2, and are finally detected by two avalanche photodiodes APD1 and APD2 (*τ*-SPAD Single Photon Counting Module, PicoQuant). We use Brightline HC 525/50, HC 600/37, HC 676/37, (all Semrock) bandpass filters for filtering 470, 561 and 640 nm laser-excited fluorescence, respectively. A TCSPC card (SPC-150, Becker & Hickl GmbH, Berlin, Germany) records, for each photon, its absolute arrival time as well as its delay time with respect to the 40 MHz laser trigger.

The spatial overlap of the foci is carefully checked in all three dimensions by using 100-nm multi-color fluorescent beads (TetraSpeck™ Microspheres, fluorescent blue/green/orange/dark red, Thermo Fisher Scientific, Darmstadt, Germany) and aligned accordingly. The lateral 1/*e*^2^ extension (*ω*_0_) of the observation volume with 470-nm, 561-nm, and 640-nm excitation is determined by measuring 3D diffusion of Atto488-carboxylic acid, Atto550 and Atto655-NHS ester in water using conventional FCS, yielding diffusional correlation times, *τ*_D_, for the different fluorophores. Next, the parameter *ω*_0_ = (4*Dτ*_D_)^1/2^ is calculated for each of the three wavelengths using published diffusion coefficients, *D*, determined by dual-focus FCS^32^. The ratio between the axial (*z*_0_) and lateral (*ω*_0_) extensions is determined by imaging 100-nm multi-color fluorescent beads. The parameters resulting from the calibration were *ω*_0_ = 0.23 ± 0.02 µm and *z*_0_ = 0.76 ± 0.03 µm for 470-nm light; *ω*_0_ = 0.25 ± 0.01 µm and *z*_0_ = 0.87 ± 0.03 µm for 561-nm light; and *ω*_0_ = 0.30 ± 0.02 µm and *z*_0_ = 1.45 ± 0.03 µm for 640-nm light. For dual-color cross-correlation, the effective values of *ω*_0_ and *z*_0_ are given by the root-mean-square averages of these parameters for two colors.

### GUV preparation and labeling

We prepared GUVs labeled with the two individual dyes Atto647N and DiI according to the protocol by García-Sáez *et al*.^22^, using a mixture of DOPC (79.995 mol%), cholesterol (19.999 mol%), Atto647N-DPPE (0.001 mol%), and DiI (0.005 mol%) (all from Sigma-Aldrich, St. Louis, MO). For labeling with a dual-color probe, we used DOPC (90 mol%) and cholesterol (10 mol%) and added a double-stranded DNA, with strand 1 carrying a cholesterol anchor for membrane insertion and the complementary strand 2 Atto488 and Atto565 fluorophores, during the last step of GUV preparation. DNA oligos were designed and ordered from IBA GmbH (Göttingen, Germany). The sequence (5′ to 3′) of strand 1 was GCA GTG CCG CAT TGG AAC AGG TAG GCT CCT CAA CCA TCC GTC CCG AGT [C], with [C] denoting the modified cytidine with cholesterol attached; the sequence of strand 2 was GGA [T]GG TTG AGG AGC CTA CCT GTT CCA ATG CGG CAC TG[C]. Here, [T] and [C] denote the thymidine and cytidine nucleotides with attached Atto488 and Atto565, respectively. Lyophilized dye- and cholesterol-labeled DNA oligo samples were dissolved in PBS (Thermo Fisher Scientific) to a concentration of 500 µM. For hybridization, 1 µL dye-labeled DNA, 2 µL cholesterol-labeled DNA, 7 µL buffer solution (100 mM NaCl (Sigma-Aldrich), 50 mM Tris (Sigma-Aldrich)) were mixed and heated to 94 °C for 2 min, followed by slow cooling for 2 h to room temperature.

### Cell culture and membrane staining

Lung cancer cells (NCI-H1703) were maintained at 37 °C and 5% CO_2_ in Roswell Park Memorial Institute (RPMI) medium supplemented with 10% fetal bovine serum and 1% sodium pyruvate (Thermo Fisher Scientific). The lsFCS and lsSCS measurements were performed 24 h after seeding the cells on 8-well Nunc Lab-Tek chambered cover glass (Thermo Fisher Scientific). For membrane staining, stock solutions of CellMask Deep Red and CellMask Green plasma membrane stains (Thermo Fisher Scientific) were diluted with pre-warmed RPMI medium at a ratio of 1 : 3 × 10^6^ and 1 : 2.5 × 10^4^, respectively. The cell medium was exchanged with the dye solution and, 10 min later, the staining medium was again replaced by fresh and pre-warmed RPMI medium. Immediately afterwards, the experiments were started.

## Electronic supplementary material

Supplementary Information
