# Supplementary material for: Pulsed interleaved excitation-based line-scanning spatial correlation spectroscopy (PIE-lsSCS)
Source: Sci Rep. 2018 Nov 13;8:16722. doi: 10.1038/s41598-018-35146-4 (PMC6233157; doi:10.1038/s41598-018-35146-4)
Supplement: Supplementary file 1 — Supplementary Information [file 41598_2018_35146_MOESM1_ESM.pdf]

Pulsed interleaved excitation-based line-scanning spatial correlation spectroscopy (PIE-lsSCS)

Xiang Gao[1, †], Peng Gao[1,2,†], Benedikt Prunsche[1], Karin Nienhaus[1], and G. Ulrich Nienhaus[1,2,3,4,*]

[1]*Institute of Applied Physics, Karlsruhe Institute of Technology, 76128 Karlsruhe, Germany*
[2]*Institute of Nanotechnology, Karlsruhe Institute of Technology, 76344 Eggenstein-Leopoldshafen, Germany*
[3]*Institute of Toxicology and Genetics, Karlsruhe Institute of Technology, 76344 Eggenstein-Leopoldshafen, Germany*
[4]*Department of Physics, University of Illinois at Urbana-Champaign, Urbana, Illinois 61801, USA*

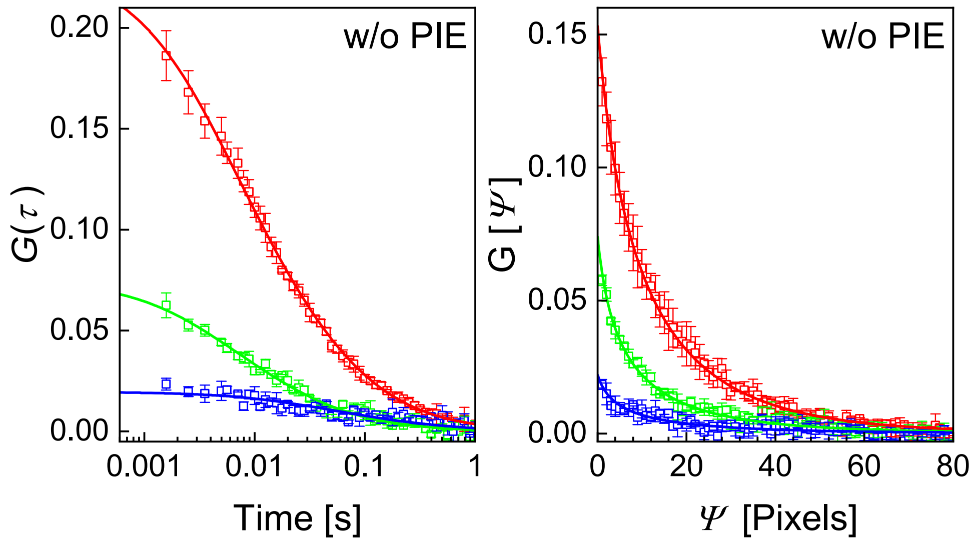

Fig. S1. lsFCS and lsSCS experiments on DOPC/cholesterol GUVs labeled with DiI and Atto647N-DPPE. The sample was excited by interleaved 561-nm and 640-nm laser pulses. The emitted fluorescence was collected in the green (600/37 nm, central/bandwidth) and red (676/37 nm) color channels. lsFCS (left) and lsSCS (right) correlation data (symbols) were calculated from intensity time traces, using all photons collected in the two channels (i.e., without time filtering as in PIE). Autocorrelation functions in the green and red channels are plotted in green and red, respectively; cross-correlation functions are shown in blue. Lines show fits with a 2D diffusion model.
